# Physical activity and hippocampal volume in young adults

**DOI:** 10.1007/s11682-024-00916-4

**Published:** 2024-09-09

**Authors:** Anastasia Cherednichenko, Anna Miró-Padilla, Jesús Adrián-Ventura, Irene Monzonís-Carda, Maria Reyes Beltran-Valls, Diego Moliner-Urdiales, César Ávila

**Affiliations:** 1 Neuropsychology and Functional Neuroimaging Group, Department of Basic Psychology, Clinical Psychology and Psychobiology, Universitat Jaume I, Castelló de la Plana, Spain; 2 Neonatal Research Group, Health Research Institute La Fe, Avenida Fernando Abril Martorell 106, Valencia, 46026 Spain; 3 Department of Psychology and Sociology, University of Zaragoza, Teruel, 44003 Spain; 4 LIFE Research Group, Department of Education, Universitat Jaume I, Castellon, 12071 Spain

**Keywords:** Physical activity (PA), Hippocampus, Punishment sensitivity, Young adults, Accelerometer, Personality, Voxel-based morphometry

## Abstract

Evidence from previous studies suggests that physical activity (PA) may contribute to functional and structural changes in the hippocampus throughout the lifespan. However, there is limited evidence available regarding the young adult population. Additionally, the personality traits that may influence this association remain unclear. With a sample of 84 young adults (43 women; age 22.7 ± 2.8y; range 18–29), the main aim of the current study was to analyze the association between objective and self-reported measures of daily PA and hippocampus subfield gray matter volumes, and to examine the role of the personality trait of punishment sensitivity in this association. Our results showed that only moderate to vigorous levels of objectively measured PA were positively associated with the hippocampal CA2/CA3 volume. Moreover, punishment sensitivity correlated negatively with the objective measure of sedentarism and with self-reported measures of PA. However, regression analyses did not find any interaction between punishment sensitivity and PA in explaining individual differences in hippocampal volumes. Thus, our data suggest that intense PA may contribute to enhancing the hippocampal CA2/CA3 volume in young adults.

## Introduction

Lifestyle behaviors tend to be established during young adulthood, forming patterns that may persist later in life, with consequential health benefits or risks (Grant et al., 2019). According to previous studies, the benefits of maintaining an active lifestyle during this period in life are currently well accepted, not only due to its benefits for physical health, but also due to its influence on memory and cognitive function (Hillman et al., 2008) and in preventing neurodegenerative decline (Blondell et al., 2014). In fact, neuroimaging studies have shown that regular physical activity (PA) can have a functional and structural influence on brain regions like the hippocampus throughout life (Erickson et al., 2019; Valkenborghs et al., 2019).

The hippocampus is one of the main brain sites of neuroplasticity, and it has been identified as highly sensitive to the effects of PA (Cotman et al., 2007). In one of the most influential studies in this field, Erickson et al. (2011) showed that 12 months of aerobic physical training increased hippocampal volume and serum brain-derived neurotrophic factor (BDNF) levels in a sample of older adults, suggesting an association whereby physical exercise promotes neurogenesis in the hippocampus, as supported by animal studies (Joseph et al., 2023). Cross-sectional studies using self-reported questionnaires have confirmed a positive association between self-reported PA and hippocampal gray matter (GM) volume in healthy adults aged 19 to 82 years (Demirakca et al., 2014; Killgore et al., 2013). However, since the study by Erickson and colleagues, research studies have provided partial support for their results. While some meta-analyses have reinforced this causal relationship (Feter et al., 2018; Firth et al., 2018; Wilckens et al., 2021) others have shown no relationship between hippocampal volume and training (Balbim et al., 2024; Wilckens et al., 2021, for healthy participants). According to these latter meta-analyses, which reviewed randomized controlled trials, the lack of this relationship was found in studies with healthy and older participants with interventions lasting more than 6 months. Thus, several factors such as the duration, type, and frequency of the interventions differed across the studies, so the study design may help explain the failure in replicability.

The inconsistent results obtained in the meta-analyses of the relationship between hippocampal volume and PA can be attributed to several factors. First, many studies have been conducted in healthy older populations or samples with pathologies. In contrast, there are few studies involving healthy young individuals. Some studies with younger samples have produced positive results (Griffin et al., 2011; Nauer et al., 2020) but a recent meta-analysis concluded that the relationship is only observed when mean age is older than 65 years (Wilckens et al., 2021). Thus, age is a relevant factor when considering the effect of PA on the hippocampus.

A second factor to consider when studying the relationship between PA and the hippocampus is the need to examine the various subdivisions of this structure separately, as not all parts are susceptible to neurogenesis. Recent studies in humans have shown that the CA4/Dentate Gyrus and CA2/CA3 regions are the most sensitive to the effects of PA. Suwabe et al. (2018) demonstrated that a single bout of exercise increases the connectivity of these areas with other cortical zones, predicting improvements in memory tests. Furthermore, randomized controlled trials with healthy participants have found that aerobic exercise training increased hippocampal volume in these areas compared to the control group (Frodl et al., 2020; Nauer et al., 2020), as well as cerebral blood flow (Pereira et al., 2007). Additionally, it has been shown that a single session of exercise initiates a cascade of neurophysiological mechanisms in these areas that could lead to neurogenesis (Callow et al., 2023). In summary, the effects of physical exercise on the hippocampus may be more evident in the CA3 and CA4/DG areas.

A third source of variability highlighted in previous cross-sectional studies is the assessment of PA: subjective methods are more susceptible to measurement error and biases than objective measures of PA (e.g. imprecise recall or influence of social desirability leading to overestimation of real PA levels) (Rhodes & Smith, 2006). This fact has led to the recommendation of using objective measurements of PA whenever possible (Downs et al., 2014). In recent years, the use of accelerometers has increased the ability to objectively measure the frequency, duration, and intensity of PA throughout the day. In this line, emerging evidence has provided new insights about the association between these accelerometer-derived PA components and hippocampal volume (Hamer et al., 2018; Spartano et al., 2019; Varma et al., 2015). Hamer et al. (2018) showed that total PA was positively associated with left and right hippocampal volume in adults with a mean age of 55.4 years old. Similarly, Spartano et al. (2019) and Varma et al. (2015) showed that light PA, such as daily walking, was correlated with an enhancement of brain volume in adults and older adults. Nonetheless, there is still not enough evidence about the PA intensity that may be necessary to enhance brain function in youth (Dowd et al., 2018; Spartano et al., 2019). Hence, a greater understanding of the association between objectively measured PA and hippocampal volume is needed.

Despite the health benefits of maintaining an active lifestyle for physical and brain health (Hillman et al., 2008), the World Health Organization has shown that 27.5% of adults do not meet the recommendation of practicing at least 150–300 min per week of moderate PA (MPA) or at least 75–150 min per week of vigorous PA (VPA) (Bull et al., 2020; Guthold et al., 2018). To date, the factors that predispose adults to maintaining an active or sedentary lifestyle are still unknown (Rhodes & Smith, 2006). Nevertheless, it is known that PA reduces anxiety (Frederiksen et al., 2021; Piva et al., 2023; Wu et al., 2022), and this effect could be influenced by personality traits (Rhodes & Smith, 2006). Among different personality traits, the punishment sensitivity dimension described in the Reinforcement Sensitivity Theory offers a good neuropsychological framework to investigate individual differences in the relationship between PA and hippocampal anxiety (McNaughton & Gray, 2000). Punishment sensitivity is a dimension that depends on the septohippocampal system, which determines the individual differences in sensitivity and reactivity to aversive stimuli. The two most popular psychometric measures of this trait are the Behavioral Inhibition scale (BIS) and the Sensitivity to Punishment (SP) scale (Torrubia et al., 2008), which are directly related to the hippocampus (Barrós-Loscertales et al., 2006; Cherbuin et al., 2008; Levita et al., 2014) and also to individual differences in anxiety (Corr, 2004). Previous studies relating these BIS measures to PA have yielded different results depending on whether self-reported or objective measures of PA were employed. When self-reported measures of PA levels were used, scores of the BIS scale correlated positively with self-reported physical inactivity (Voigt et al., 2009). This result is consistent with a previous meta-analysis showing that Neuroticism and related measures predispose individuals to self-report lower levels of physical activity (Sutin et al., 2016; Wilson & Rhodes, 2021). However, when objective measures of PA were used, the relationship of BIS and Neuroticism scores with PA was negative (Wilson & Dishman, 2015; Kekäläinen et al., 2020). Thus, previous results suggest that individuals with higher scores on BIS-related measures tend to subjectively underestimate their amount of PA.

Given the previous scientific literature, as well as the scarce research using objective measures of PA in the young adult population, the main aims of the current study were: (i) to analyze the association between self-reported and objectively measured PA and the CA2/3 and CA4/DG hippocampal GM volume in a sample of young adults (under 30 years old), and (ii) to examine the association of SP and BIS in this relationship. Given the positive associations between PA and hippocampal volume in prior studies (Hamer et al., 2018; Spartano et al., 2019; Varma et al., 2015) and the specificity of the effects to some hippocampal subfields (Suwabe et al., 2018; Frodl et al., 2020 Nauer et al., 2020), we expected to find a higher CA2/3 and CA4/DG hippocampal volumes among physically active young adults. Moreover, because previous data suggested that BIS measures were positively associated with hippocampal volume, we expected to find that this trait contributes to the relationship between PA and hippocampal CA2/3 and CA4/DG volumes.

## Materials and methods

### Participants

A total of 84 healthy young adults (43 women), with a mean age of 22.7 years (*SD* = 2.8 years, range 18–29), participated in the study after signing an informed written consent. They were recruited from the student community of Universitat Jaume I through poster advertisements, social networks, and word of mouth. All the participants met the criteria of being right-handed and having achieved a minimum scaled score of 7 on the Matrix Reasoning subtest of the Wechsler Adult Intelligence Test Scale, 3^rd^ edition (WAIS-III), indicating adequate intellectual functioning. Most of the participants (86.9%) had a tertiary education level, such as a bachelor’s degree (67.9%) or master’s/ doctoral degree (19%). Furthermore, none of them reported a previous history of psychiatric or neurological disorders or brain injury with loss of consciousness. The research project was approved by the Ethical Committee of Universitat Jaume I (CD/11/2021), and all the volunteers received monetary compensation for taking part in the study.

### PA measurement

#### Self-reported PA

Self-reported measures of PA levels were obtained via Qualtrics through the Spanish version of the self-reported Global Physical Activity Questionnaire (GPAQ), published by the World Health Organization as part of the WHO STEPwise Approach to Noncommunicable Disease Risk Factor Surveillance. Data were analyzed using GPAQ analysis guidelines, which include 16 items designed to assess duration (minutes/day), frequency (days/week) and intensity (moderate and vigorous) of PA during a typical week. Activities are classified into three intensity levels based on the metabolic equivalent of task (MET), commonly used to express the intensity of physical activities. Moderate and vigorous METs were calculated and then combined to obtain the self-reported Total PA. Three participants did not adequately complete the GPAQ and were excluded from the analyses.

#### Objectively measured PA

Objective measures of PA levels were obtained using the GENEActiv accelerometer (Activinsights Ltd, Kimbolton, UK), a waterproof device with a triaxial microelectromechanical sensor that records both motion-related and gravitational acceleration and has linear and equal sensitivity along the three axes. The device also includes a body temperature sensor to help confirm wear and non-wear time and provides a reliable (coefficient of variation intra and inter instrument of 1.4% and 2.1%, respectively) and valid assessment of PA in young adults (Esliger et al., 2011). Accelerometers were programmed with a frequency sampling of 100 Hz, and data were stored in gravity (g) units (1 g = 9.81 m/second^2^). The raw acceleration output was converted to 1-second epochs using the GENEActiv Post-Processing PC Software (version 2.2, GENEActiv). Participants wore the accelerometer on their non-dominant (left) wrist during seven consecutive days prior to the magnetic resonance imaging (MRI) session, with 24-hour valid data. Thus, by combining all the registered days for each participant, PA was expressed as the average (min/day) sedentary time, light physical activity (LPA), and moderate/vigorous physical activity (MVPA: the sum of MPA and VPA). A total of 15 devices failed to register the PA at some point during the 7 days and were not considered for the analyses.

### Personality traits

Two different measures of punishment sensitivity and anxiety-proneness bases on the RST were administered. The Sensitivity to Punishment and Sensitivity to Reward Questionnaire (SPSRQ; Torrubia et al., 2001) was used to assess individual differences in SP. The SPSRQ is a 48-item, yes–no questionnaire consisting of two scales: SP (24 items) and Sensitivity to Reward (SR; 24 items). This questionnaire measures individual differences in sensitivity to various punishment and rewards, describing heterogeneous situations that elicit responses to obtain punishments or rewards. The SP score was obtained by adding all the positive “yes” responses. The BIS scale of the BIS/BAS questionnaire (Carver & White, 1994) was also used to measure punishment sensitivity. This is a 7-item self-report scale that measures the motivation to avoid aversive outcomes. Participants respond to each item using a 4-point Likert scale from “very true for me” to “very false for me”.

### Magnetic resonance imaging acquisition

MRI scanning sessions for anatomical brain data acquisition were performed using a 3T General Electric Signa Architect scanner (Waukesha, WI, USA). A high-resolution T1-weighted BRAVO sequence was acquired, covering the whole brain (TE = 3.28 ms, TR = 8.52 ms, FOV = 240 mm, phase FOV = 100%, flip angle = 12º, inversion time = 450 ms, matrix = 256 × 256, voxel size = 0.5 × 0.5 mm, space between slices = 0.5 mm, slice thickness = 1 mm, number of images = 384, sequence length = 4:17 min). A 24-channel coil was used, and the scan plane angulation was strictly sagittal. Inside the MRI scanner, volunteers were placed in a supine position, and their heads were immobilized with cushions to reduce involuntary movement. For a more comfortable and enjoyable experience during the sequence, landscape images were presented through MRI-compatible goggles (VisuaStim, Resonance Technology Inc., Northridge, CA, USA).

### Image preprocessing

First, the anatomical T1-weighted images were aligned to the anterior–posterior commissure line. Subsequently, voxel-based morphometry analysis was performed using the Computational Anatomy Toolbox (CAT12; v12.8, r1885; www.neuro.uni-jena.de/cat/*)* for the Statistical Parametric Mapping software (SPM12; v7771; www.fil.ion.ucl.ac.uk/spm/software/spm12/*)* under Matlab R2018b (v9.5). Using the CAT12 standard “segment” module, all the steps recommended in the manual were performed: (1) segmentation of the images into GM, white matter (WM), and cerebrospinal fluid (CSF); (2) registration and regularization to a standard template provided by the International Consortium for Brain Mapping (ICBM); (3) DARTEL normalization of all the GM segments to the Montreal Neurological Institute (MNI) template; and (4) modulation by the affine + nonlinear components (SPM default) derived from the spatial MNI normalization. After this preprocessing pipeline, the total intracranial volume (TIV) was estimated as the sum of the GM, WM, and CSF volumes. Afterwards, a data quality check of the resulting images was conducted using the CAT12 “check sample homogeneity” module, which showed no potential outliers in brain volume.

### Data analyses

First, descriptive analyses (shown in Table 1) were performed for all variables, and normal data distribution was examined using the Kolmogorov-Smirnov test. Second, region of interest (ROI) analyses were conducted following the CAT12 standard pipeline (“ROI Tools” module). To do this, we first extracted volumetric data bilaterally from the hippocampal formation (subiculum, molecular layer, CA1, CA2/3, CA4/DG) using the Cobra Atlas (Winterburn et al., 2013; https://www.cobralab.ca/atlases) provided in the CAT12. This strategy allowed us to obtain the GM volume (in ml) of the five hippocampal subfields for each participant in native space. Afterwards, volumetric, personality, and PA (objective and self-reported) data were analyzed using IBM SPSS Statistics for Windows, Version 28.0 (Armonk, NY: IBM Corp) through partial correlations (controlling for age and total GM volume). A Bonferroni correction for multiple comparisons, including five hippocampal subfields and five PA measures (α = 0.05/25 = 0.002), was applied to the study of the relationship between hippocampal volume and PA. This correction was not applied to the study of the relationship between punishment sensitivity and PA, as it was hypothesis-driven. In addition, hierarchical multiple regressions were performed to determine whether PA and personality traits predicted the volume of the hippocampus, using the different ROIs as dependent variables. In the first step, age and total GM volume were introduced into the model using the enter method. In the second step, SP and BIS measures, as well as objective and subjective PA measures, were introduced into the model using the stepwise method. The stepwise method was adopted to extract only significant (*p* < .05) predictors. Finally, to investigate the potential effects of sex on our results, we conducted sex-stratified analyses.

**Table 1:** Descriptive characteristics of the study sample

	Men (*N* = 41)	Women (*N* = 43)	All (*N* = 84)				
Age (years)	23.2 ± 3.1	22.2 ± 2.5	22.7 ± 2.8				
**Personality measures**							
Sensitivity to Punishment	10.2 ± 5.2	12.3 ± 5.1	11.3 ± 5.2				
Behavioral Inhibition scale	19.8 ± 3.0	23.3 ± 2.5	21.6 ± 3.3				
**Brain Volumes (ml)**							
Total GM volume	784.35 ± 53.58	701.73 ± 45.87	742.06 ± 64.61				
Bilateral CA1	1.75 ± 0.15	1.61 ± 0.15	1.68 ± 0.17				
Bilateral CA2/CA3	0.34 ± 0.04	0.32 ± 0.03	0.33 ± 0.04				
Bilateral CA4/Dentate Gyrus	1.83 ± 0.17	1.72 ± 0.16	1.78 ± 0.17				
Bilateral Stratum	1.26 ± 0.12	1.16 ± 0.11	1.21 ± 0.12				
Bilateral Subiculum	0.63 ± 0.06	0.56 ± 0.06	0.60 ± 0.06				
**Self-reported PA level (METs; ** *N* ** = 81)**							
Total	4192.9 ± 3549.2	4342.4 ± 3857.1	4270.4 ± 3689.7				
**Objective PA levels (min/day) (** *N* ** = 69)**							
Sedentary time	802.2 ± 75.8	745.3 ± 58.7	774.1 ± 73.1				
Light	138.8 ± 46.0	165.9 ± 50.8	152.2 ± 49.9				
Moderate and Vigorous	51.6 ± 20.6	59.7 ± 21.8	55.6 ± 21.4				

Note. Values are mean ± standard deviation or frequency (%). Hippocampal subfield volumes were computed bilaterally

## Results

Descriptive characteristics of the study sample are shown in Table 1. From the initial sample (*N* = 84), a total of 81 participants (42 women, age = 22.7 ± 2.8 years) with valid self-reported PA data and 69 participants (34 women, age = 22.7 ± 2.8) with valid objectively measured PA data were included in the analyses. Objectively measured PA registered during seven consecutive days prior to the MRI session, revealed that participants spent, on average, 152.2 min per day on LPA, and 55.6 min per day on MVPA.

### Whole sample analyses

First, we calculated partial correlations, controlling for total GM volume and age, between personality measures and hippocampal subfield volumes. All of these correlations were non-significant (*p > .10*). The correlations of objective and self-reported PA with personality traits and hippocampal volumes are shown in Table 2. Regarding the analyses between PA measurements and brain volume, non-parametric partial correlations between objectively measured data and hippocampal GM volume revealed a positive association of MVPA with the GM volume of the CA2/CA3 area of the hippocampus. The correlation of the CA2/CA3 volume with LPA and between CA4/DG and MVPA were significant at *p* < .05, but did not survive after correction for multiple comparisons. There were not significant correlations between any other objective measure of PA levels and the selected ROIs. Concerning the self-reported PA measure, the analyses did not show any significant correlation with hippocampal volumes.

**Table 2:** Spearman’s correlations and partial correlations between objective and self-reported levels of PA, personality trait, and hippocampal volumes

		Objectively measured PA (min/day) (*N* = 69)			Self-reported PA (METs) (*N* = 81)	
		Sedentary	Light	Moderate & Vigorous	Total	
**Punishment Sensitivity** ^**$**^	SP	− 0.07 *P* = .55	0.04 *P* = .73	− 0.08 *P* = .54	− 0.32** *P* = .007	
	BIS	− 0.37** *P* = .002	0.12 *P* = .31	0.02 *P* = .95	− 0.28* *P* = .01	
**Hippocampus volume** ^**$$**^	CA1	0.04 *P* = .76	0.00 *P* = .98	0.03 *P* = .78	-0.04 *P* = .72	
	CA2/ CA3	0.02 *P* = .86	0.27* *P* = .015	0.35** *P* = .002	− 0.07 *P* = .52	
	CA4/DG	0.01 *P* = .92	0.16 *P* = .09	0.21* *P* = .05	− 0.07 *P* = .54	
	Subiculum	− 0.07 *P* = .56	− 0.15 *P* = .22	− 0.12 *P* = .32	0.07 *P* = .52	
	Stratum	0.01 *P* = .94	0.07 *P* = .28	0.12 *P* = .33	− 0.12 *P* = .28	

^$^ Spearman’s correlations; ^$$^ Non-parametric partial correlations controlling for total GM and age; *p < .05; **P corrected for multiple comparisons

SP: Sensitivity to Punishment; BIS: Behavioral Inhibition System

On the other hand, significant correlations were found between personality and PA levels. Specifically, the analyses showed a negative correlation between the BIS scale and sedentary time when measured objectively. Likewise, a significant negative correlation was also found between the BIS scale and self-reported Total PA. Moreover, self-reported Total PA also showed significant negative correlations (*p* < .01) with the SP scale.

Finally, several multiple regression analyses were conducted to examine possible additive effects of the PA measures and personality on GM hippocampal volumes. We did not obtain any significant interaction between these variables in any of the models.

### Sex-stratified analyses

To explore the potential contribution of sex to our findings, we conducted sex-stratified analyses (see Table 3). Apart from the correlation between BIS and sedentarism, observed solely among men, all other effects exhibited consistent contributions in the same direction for both sexes, which in turn demonstrates the effect of the overall sample.

**Table 3:** Spearman’s correlations and non-parametric partial correlations between objective and self-reported levels of PA, personality trait, and hippocampal volumes stratified by sex

Men		Objectively measured PA (min/day) (*N* = 35)			Self-reported PA (METs) (*N* = 41)	
		Sedentary	Light	Moderate & Vigorous	Total	
**Punishment Sensitivity** ^**$**^	SP	− 0.21	0.07	− 0.01	− 0.31*	
	BIS	− 0.31*	0.03	− 0.00	-0.30*	
**Hippocampus volume** ^**$$**^	CA1	0.27	0.03	− 0.01	− 0.12	
	CA2/ CA3	0.18	0.24	0.34*	− 0.05	
	CA4/DG	0.15	0.09	0.24	− 0.06	
	Subiculum	- 0.04	− 0.14	− 0.07	0.06	
	Stratum	0.10	0.05	0.20	− 0.25	
**Women**		**Objectively measured PA (min/day)** (*N* = 34)			**Self-reported PA (METs)** (*N* = 43)	
		Sedentary	Light	Moderate & Vigorous	Total	
**Punishment Sensitivity** ^**$**^	SP	0.08	− 0.13	− 0.25	− 0.32*	
	BIS	− 0.01	− 0.09	− 0.33*	-0.34*	
**Hippocampus volume** ^**$$**^	CA1	- 0.03	− 0.11	− 0.05	0.02	
	CA2/ CA3	− 0.06	0.39*	0.40*	− 0.09	
	CA4/DG	− 0.08	0.09	0.12	− 0.03	
	Subiculum	− 0.04	− 0.21	− 0.24	0.09	
	Stratum	− 0.04	0.03	0.00	0.03	

^$^ Spearman’s correlations; ^$$^ Non-parametric partial correlations controlling for total GM and age; *p < .05; **P corrected for multiple comparisons

SP: Sensitivity to Punishment; BIS: Behavioral Inhibition System

## Discussion

The main finding of the present study indicates that daily objectively measured MVPA was positively associated with the hippocampal CA2/CA3 volume, and that sedentarism was negatively associated with punishment sensitivity in young adults. Moreover, regarding self-reported PA, our results revealed that these relationships are observed only with personality traits, but not with the hippocampal volumes. These results contribute to the current scientific knowledge by suggesting that there is an association between MVPA and hippocampal volume in young adults, but no interaction with BIS-related measures.

The results of our study seem to be partially in line with previous research analyzing the association between PA and hippocampal volume in older populations, where MPA and LPA were positively associated with brain volume (Varma et al., 2015; Hamer et al., 2018; Spartano et al., 2019), and with randomized controlled trials showing that exercise training was associated with increased hippocampal volume (Feter et al., 2018; Firth et al., 2018; Wilckens et al., 2021). In our study with a sample of young adults, we only found a positive relationship between hippocampal CA2/CA3 volume and MVPA. This PA intensity is prevalent in sports and gym activities, which are more common among youths than older adults, whereas LPA is more often observed in older adults’ activities of daily living, such as self-care or walking. Thus, our results suggest that the impact of PA on hippocampal volume is more clearly observed after MVPA. Additionally, we have obtained correlations that did not survive multiple comparisons: LPA with CA2/CA3 volume and MVPA with CA4/DG volume.

Recent evidence suggests that CA2/CA3 is involved in the representation of sequentially presented events in the same context but not in generalization processes (Bein & Davachi, in press). In contrast, the role of CA4/DG seems to be more related to temporal differentiation of events and therefore, in generalization processes. Since accelerometer recording is done continuously over seven days during 24h, we can assume that moments requiring moderate to vigorous PA are moments of high motivation and drive, thus enhancing episodic memory of the that event. Therefore, we speculate that the activity and volume in CA2/CA3 could increase with intense physical exercise to efficiently encode such episodes. This result aligns with the literature in humans showing that acute PA promotes specific modifications in these hippocampal subregions (Suwabe et al., 2018; Frodl et al., 2020; Nauer et al., 2020) and with animal literature showing that PA induces neurogenesis, angiogenesis, and neuroplasticity due to increases in the release of neurotransmitters and neurotrophic factors (Matta Mello Portugal et al., 2013) in these specific brain regions (Hillman et al., 2008), which may lead to better memory and cognitive function (Erickson et al., 2019).

Based on this study, we can infer the nature of the relationship between physical training and hippocampal volume. The fact that objective accelerometer measures taken in the days preceding the scanner session were more strongly associated with hippocampal volume than the self-reported measure suggests that the effect may be acute rather than chronic. In this sense, the weekly estimation does not clearly determine the nature of the effect, as it may reflect the participant’s habitual activity. Future studies should investigate the possibility that the observed brain changes were due to activity performed in the last few days rather than a chronic effect.

Regarding personality, our results suggest that self-reported and accelerometer-derived measures of PA and sedentarism were significantly associated with scores on the SP and BIS scales. Importantly, these measures exhibit opposing trends. The negative association between self-reported measures and anxiety-related scales has been consistently reported in several meta-analyses (Rhodes & Smith, 2006; Wilson & Dishman, 2015; Allen et al., 2019). The novelty of our study lies in the objective measure of sedentarism, which correlates negatively with the BIS scale for males. Thus, anxiety-proneness appears to be linked to a stronger perception of being less active but engaging in less sedentary behavior when objectively measured. A recent meta-analysis analyzing the relationship between sedentarism and anxiety symptoms found a positive and moderate association between the two factors when assessed using self-reported measures (Stanczykiewicz et al., 2019). However, recent studies utilizing accelerometer-derived measures did not find any relationship between individual differences in neuroticism and sedentarism (Hearon & Harrison, 2021; Kekäläinen et al., 2020). In the study by Kekäläinen et al., the anxiety facet of Neuroticism was associated with a greater discrepancy between subjective measures and accelerometer-measured activity. Nonetheless, given our study’s results and considering that most publications included older individuals in the meta-analysis employing subjective reports of PA, generalizing this relationship may be premature. Although not the primary focus of our study, the potential for disparate results depending on the measure type offers a fresh perspective on this effect.

### Limitations and strengths

The main strengths of our study comprise the inclusion of self-reported and objective PA with high-wear time compliance, ensuring all participants had seven days of valid data. Another important strength of this study is its novelty, as it is the first study to investigate the role of BIS-related measures in the association between daily PA intensity levels and hippocampal volume. However, our results should be interpreted with caution because these analyses were conducted on a relatively small sample, which limits the generalizability of our findings. Additionally, the cross-sectional design of our analyses prevents us from inferring causal relationships. While we suggest that PA could contribute to the enhancement of hippocampal volume, recent data have also suggested that cognitive decline may lead to reduced PA (Sabia et al., 2017).

## Conclusions

The current study showed that objectively measured PA is positively associated with hippocampal CA2/CA3 volume in young adults. The pattern of relationships between PA and punishment sensitivity revealed a negative relationship between both variables when using self-reported measures of PA, but an inverse relationship with accelerometry-derived sedentarism measure. Thus, our findings contribute to the current scientific knowledge and add new evidence, particularly in the young adult population.

## Data Availability

The data that support the findings of this study are available upon reasonable request.
